# Machine Learning Decision Tree Models for Differentiation of Posterior Fossa Tumors Using Diffusion Histogram Analysis and Structural MRI Findings

**DOI:** 10.3389/fonc.2020.00071

**Published:** 2020-02-07

**Authors:** Seyedmehdi Payabvash, Mariam Aboian, Tarik Tihan, Soonmee Cha

**Affiliations:** ^1^Department of Radiology and Biomedical Imaging, Yale School of Medicine, New Haven, CT, United States; ^2^Department of Radiology and Biomedical Imaging, University of California, San Francisco, San Francisco, CA, United States; ^3^Department of Pathology, University of California, San Francisco, San Francisco, CA, United States

**Keywords:** posterior fossa tumor, diffusion histogram, machine learning, decision tree model, MRI

## Abstract

We applied machine learning algorithms for differentiation of posterior fossa tumors using apparent diffusion coefficient (ADC) histogram analysis and structural MRI findings. A total of 256 patients with intra-axial posterior fossa tumors were identified, of whom 248 were included in machine learning analysis, with at least 6 representative subjects per each tumor pathology. The ADC histograms of solid components of tumors, structural MRI findings, and patients' age were applied to construct decision models using Classification and Regression Tree analysis. We also compared different machine learning classification algorithms (i.e., naïve Bayes, random forest, neural networks, support vector machine with linear and polynomial kernel) for dichotomized differentiation of the 5 most common tumors in our cohort: metastasis (*n* = 65), hemangioblastoma (*n* = 44), pilocytic astrocytoma (*n* = 43), ependymoma (*n* = 27), and medulloblastoma (*n* = 26). The decision tree model could differentiate seven tumor histopathologies with terminal nodes yielding up to 90% accurate classification rates. In receiver operating characteristics (ROC) analysis, the decision tree model achieved greater area under the curve (AUC) for differentiation of pilocytic astrocytoma (*p* = 0.020); and atypical teratoid/rhabdoid tumor ATRT (*p* = 0.001) from other types of neoplasms compared to the official clinical report. However, neuroradiologists' interpretations had greater accuracy in differentiating metastases (*p* = 0.001). Among different machine learning algorithms, random forest models yielded the highest accuracy in dichotomized classification of the 5 most common tumor types; and in multiclass differentiation of all tumor types random forest yielded an averaged AUC of 0.961 in training datasets, and 0.873 in validation samples. Our study demonstrates the potential application of machine learning algorithms and decision trees for accurate differentiation of brain tumors based on pretreatment MRI. Using easy to apply and understandable imaging metrics, the proposed decision tree model can help radiologists with differentiation of posterior fossa tumors, especially in tumors with similar qualitative imaging characteristics. In particular, our decision tree model provided more accurate differentiation of pilocytic astrocytomas from ATRT than by neuroradiologists in clinical reads.

## Introduction

The current standard of care for patients presenting with posterior fossa tumors is maximal safe resection of tumor, decompression to eliminate mass effect, and radiochemotherapy. While histopathological evaluation is currently the gold standard for brain tumors diagnosis, there is growing body of evidence that combination of quantitative imagining and machine learning algorithms can help with non-invasive differentiation of brain neoplasms based on pre-treatment MRI (1, 2). An accurate presurgical diagnosis can play an important role in surgical planning, determining the extent of resection (3, 4), evaluating the need for neoadjuvant therapy, defining radiation therapy field, and counseling patients and their families (5).

The apparent diffusion coefficient (ADC) values are reflective of tumor cellularity, and help with diagnostic and prognostic assessment of posterior fossa tumors (6, 7). Recent studies demonstrate the added value of quantitative diffusion analysis in differentiation of posterior fossa tumors, besides conventional structural MRI findings such as peritumoral edema, enhancement pattern, location, or extension through the foramina of Luschka/Magendie (8–10). However, prior studies were limited by restricting their analysis pool to select tumor types, analyzing few ADC quantitative metrics (e.g., ADC means, median, or minimum), or only evaluating ADC values on a single slice, thus not accounting for tumor heterogeneity (11, 12).

In current study, we assessed the volumetric voxel-based ADC histogram analysis of the tumor solid components in a large sample of posterior fossa neoplasms. Using machine learning algorithms, we utilized clinical variables, quantitative ADC histogram metrics, and qualitative MRI imaging features extracted by 2 neuroradiologists on presurgical MRI to devise decision trees for accurate diagnosis of posterior fossa tumors. We chose unequivocal imaging metrics, which can be reliably assessed on widely available image viewer software in all hospitals, and thus can be readily used in neuroradiology and neuro-oncology practices. We also compared different machine learning classification models for differentiation of the most common posterior fossa tumors, which presents as a challenge in clinical practice. Including a large number of patients with a variety of pathologies allowed us to devise comprehensive differentiation models that represent a broad range of tumor types and imitate the real-world practice in a tertiary referral center.

## Methods

### Patients' Characteristics

Clinical and imaging records of all patients with posterior fossa tumor and surgical pathology results, between January 200 Re reviewer's comment #and December 2015 at our institution, were reviewed. Patients were included if they had (1) intra-axial or intra-ventricular posterior fossa tumor, (2) surgical pathology diagnosis of a neoplasm (Table 1), and (3) a presurgical MRI including ADC map, T2-weighted, Fluid Attenuated Inversion Recovery (FLAIR), and post contrast T1-weighted sequences. The exclusion criteria were pathological diagnosis other than a malignant process (e.g., cavernoma), extra-axial location except intraventricular tumors, and an ADC map quality precluding histogram analysis. In addition, tumor pathologies with <6 subjects in our cohort were excluded from univariate, and machine learning analyses (i.e., Choroid plexus papilloma, *n* = 4; Rosette-forming glioneuronal tumor, *n* = 2; Ganglioglioma, *n* = 1; Anaplastic pleomorphic xanthoastrocytoma, *n* = 1). The Institutional Review Board approved the study design, granting a waiver of informed consent given the retrospective nature of study.

**Table 1:** List of (intra-axial/intra-ventricular) posterior cranial fossa neoplasms (*n* = 256).

**Surgical pathology diagnosis**	**Patients number (frequency)**
Metastasis	65 (25.4%)
Hemangioblastoma	44 (17.2%)
Pilocytic astrocytoma	43 (16.8%)
Ependymoma	27 (10.5%)
Medulloblastoma	26 (10.2%)
Low grade glioma/astrocytoma	10 (3.9%)
Lymphoma	8 (3.1%)
Anaplastic astrocytoma	7 (2.7%)
Atypical teratoid/rhabdoid tumor	6 (2.3%)
Glioblastoma multiforme	6 (2.3%)
Subependymoma	6 (2.3%)
Choroid plexus papilloma	4 (1.6%)
Rosette-forming glioneuronal tumor	2 (0.8%)
Ganglioglioma	1 (0.4%)
Anaplastic pleomorphic xanthoastrocytoma	1 (0.4%)

### MRI Acquisition

The presurgical MRI was performed on 1.5 and 3 Tesla MRI scanners using surgical navigation (BrainLab) imaging protocol—which included axial 2D T1 weighted images, axial diffusion-weighted images (DWI), 3D T2 weighted images, 3D FLAIR, axial susceptibility weighted imaging, dynamic contrast enhancement perfusion, and 3D post contrast T1 sequences. In majority of patients, spin-echo echo-planar DWI was performed in 2D axial plane on a GE Discovery MR750 3T scanner (Waukesha, WI), with image acquisition at b = 0 s/mm^2^ and b = 1,000 s/mm^2^; repeat time = 8,300 ms, echo time = 65 ms, section thickness of 2 mm, field of view of 250 mm, and matrix size of 128 × 128.

### Qualitative Assessment of Posterior Fossa Tumors

**Figure 1:**
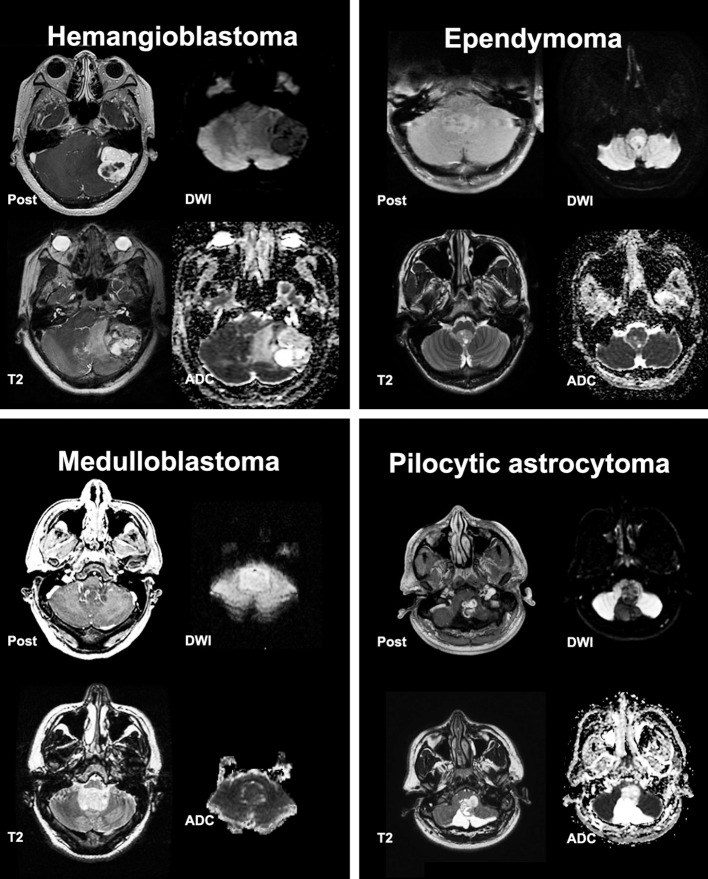
Post contrast T1, T2, Diffusion Weighted Imaging (DWI) and Apparent Diffusion Coefficient (ADC) scans from representative posterior cranial fossa tumors.

All MRI scans were reviewed independently by two board-certified neuroradiologists (SP and MA), each with 8 years of experience in interpretation of brain tumor MRI. Except for the patients' age, the reviewers were blinded to clinical information, radiology report, and pathological diagnosis at the time of review. Both SP and MA predicted the single most likely differential diagnosis for each tumor based on presurgical brain MRI. In addition, the official “clinical report” in the electronic medical records were examined to identify the foremost differential diagnosis in the “impression,” which considered as the most likely diagnosis for comparison purposes. In addition, the imaging characteristics—listed in Table 2—were extracted and corroborated with the official clinical report. In case of discrepancy between the neuroradiologist reviewers and official clinical report, the senior author (SC) reviewed the scan to reach consensus. The lesion morphology was categorized as predominantly solid (>80% solid component), mixed solid and cystic, cystic/necrotic (>80%) with irregular wall, and cystic (>80%) with smooth mural nodule. A “T2 hyperintense” solid component was determined by T2 signal greater than the gray matter (Figure 1) (13). The presence of prominent vascular flow void was assessed on T2-weighted images and confirmed on post contrast series. The tumor volumes, including both solid and cystic components, were calculated after manual segmentation on post-contrast T1 images with attention to T2/FLAIR series for non-enhancing component. We also measured the maximum radial width of FLAIR hyperintensity surrounding the tumor on axial slices as a surrogate for peritumoral edema.

**Table 2:** Structural MRI findings and clinical characteristics among various posterior fossa neoplasms.

	**MET** ** (*n =* 65)**	**HB** ** (*n =* 44)**	**PA** ** (*n =* 43)**	**EP** ** (*n =* 27)**	**MB** ** (*n =* 26)**	**LGG** ** (*n =* 10)**	**LYM** ** (*n =* 8)**	**AA** ** (*n =* 7)**	**ATRT** ** (*n =* 6)**	**GBM** ** (*n =* 6)**	**SEP** ** (*n =* 6)**	***P*-value**
**Patients' characteristics**												
Age (years)	57.6 ± 12.2	49.3 ± 17.9	18.7 ± 11.2	26.1 ± 20.1	21.8 ± 16.8	35.6 ± 27.9	63.2 ± 12.9	36.1 ± 23.7	1.3 ± 1.0	31.7 ± 22.3	50.2 ± 13.3	**<0.001**
Gender (male)	18 (33%)	19 (49%)	11 (61%)	2 (22%)	3 (50%)	2 (40%)	1 (25%)	2 (22%)	3 (50%)	2 (40%)	1 (25%)	0.304
**Tumor localization**												
Cerebellar hemisphere	54 (83%)	39 (89%)	18 (42%)	3 (11%)	9 (35%)	5 (50%)	6 (75%)	1 (14%)	2 (33%)	4 (67%)	0 (0%)	**<0.001**
Fourth ventricle	3 (5%)	3 (7%)	13 (30%)	21 (78%)	17 (65%)	0 (0%)	1 (13%)	0 (0%)	4 (67%)	0 (0%)	6 (100%)	**<0.001**
Vermis/midline	4 (6%)	2 (5%)	10 (23%)	2 (7%)	0 (0%)	0 (0%)	0 (0%)	0 (0%)	0 (0%)	0 (0%)	0 (0%)	**0.013**
Brainstem	4 (6%)	0 (0%)	2 (5%)	1 (4%)	0 (0%)	5 (50%)	1 (13%)	6 (86%)	0 (0%)	2 (33%)	0 (0%)	**<0.001**
Cerebellar peduncle involvement	4 (6%)	6 (14%)	4 (9%)	4 (15%)	5 (19%)	5 (50%)	1 (13%)	4 (57%)	2 (33%)	4 (67%)	0 (0%)	**<0.001**
**Lesion morphology**												
Predominantly solid (>80%)	46 (71%)	6 (14%)	7 (16%)	13 (48%)	17 (65%)	6 (60%)	8 (100%)	6 (86%)	3 (50%)	4 (67%)	6 (100%)	**<0.001**
Mixed solid and cystic	6 (9%)	12 (27%)	20 (47%)	14 (52%)	9 (35%)	4 (40%)	0 (0%)	1 (14%)	3 (50%)	1 (17%)	0 (0%)	**<0.001**
Cystic (>80%) with mural nodule	4 (6%)	26 (59%)	15 (35%)	0 (0%)	0 (0%)	0 (0%)	0 (0%)	0 (0%)	0 (0%)	0 (0%)	0 (0%)	**<0.001**
Necrotic with irregular wall	9 (14%)	0 (0%)	1 (2%)	0 (0%)	0 (0%)	0 (0%)	0 (0%)	0 (0%)	0 (0%)	1 (17%)	0 (0%)	**0.013**
**Enhancement pattern**												
Homogenous enhancement	16 (25%)	39 (89%)	8 (19%)	0 (0%)	5 (19%)	0 (0%)	5 (62%)	1 (14%)	0 (0%)	0 (0%)	1 (17%)	**<0.001**
Heterogeneous enhancement	49 (75%)	5 (11%)	35 (81%)	26 (96%)	20 (77%)	6 (60%)	3 (38%)	4 (57%)	6 (100%)	5 (83%)	5 (83%)	**<0.001**
No enhancement	0 (0%)	0 (0%)	0 (0%)	1 (4%)	1 (4%)	4 (40%)	0 (0%)	2 (29%)	0 (0%)	1 (17%)	0 (0%)	**<0.001**
**Extension along the neuroaxis**												
Multiple lesions	16 (25%)	4 (9%)	2 (5%)	0 (0%)	2 (8%)	1 (10%)	2 (25%)	1 (14%)	0 (0%)	2 (33%)	0 (0%)	**0.016**
Leptomeningeal drop metastasis	5 (8%)	0 (0%)	1 (2%)	0 (0%)	1 (4%)	1 (10%)	0 (0%)	0 (0%)	0 (0%)	1 (17%)	0 (0%)	0.354
**T2/FLAIR findings**												
Prominent vascular flow voids	2 (3%)	25 (57%)	0 (0%)	3 (11%)	6 (23%)	0 (0%)	0 (0%)	0 (0%)	2 (33%)	0 (0%)	0 (0%)	**<0.001**
Surrounding FLAIR (cm)	1.8 ± 0.9	1.7 ± 0.9	0.5 ± 0.5	0.4 ± 0.6	0.7 ± 0.6	0.5 ± 0.4	1.8 ± 0.6	1.0 ± 1.0	0.4 ± 0.3	1.0 ± 0.9	0.1 ± 0.1	**<0.001**
T2 hyperintense solid component^*^	9 (14%)	17 (39%)	33 (77%)	10 (37%)	6 (23%)	10 (100%)	1 (13%)	7 (100%)	0 (0%)	1 (17%)	0 (0%)	**<0.001**
**Mass effect**												
Volume (mL)	11.4 ± 9.3	19.8 ± 15.5	29.7 ± 30.1	21.6 ± 18.0	27.4 ± 19.8	14.2 ± 13.8	10.6 ± 9.1	21.4 ± 12.3	42.8 ± 35.6	12 ± 9.0	4.8 ± 4.3	**<0.001**
Hydrocephalus	22 (34%)	23 (52%)	27 (63%)	20 (74%)	19 (73%)	5 (50%)	2 (25%)	4 (57%)	5 (83%)	2 (33%)	0 (0%)	**<0.001**

*Results of univariate comparison between different neoplasm types*.

* *A “T2 hyperintense” solid component was determined by T2 signal greater than the gray matter (13)*.

*AA, anaplastic astrocytoma; ATRT, atypical teratoid/rhabdoid tumors; EP, Ependymoma; GBM, glioblastoma multiforme; HB, hemangioblastoma; LGG, low-grade glioma/astrocytoma; LYM, lymphoma; MB, medulloblastoma; MET, metastasis; PA, pilocytic astrocytoma; SEP, subependymoma*.

*A p-value < 0.05 was considered statistically significant, and depicted in bold*.

### ADC Histogram Analysis

On a GE Advantage Workstation (GE healthcare, Milwaukee, WI), we manually segmented the solid component of tumors on ADC maps with attention to post-contrast T1-weighted, T2-weighted, and FLAIR imaging. The volumetric voxel-based ADC histograms of the solid component were calculated and normalized to the average ADC value from cerebrospinal fluid in the body of lateral ventricles, as described previously (8). For each tumor, a total of 24 histogram metrics were calculated—including 21 ADC percentile values with 5 percentile increments (i.e., minimum, 5th, 10th, 15th percentile…) as well as the mean, kurtosis, and skewness. The schematic mean ADC histograms of different tumor types were developed for visual comparison.

### Decision Tree Model

**Figure 2:**
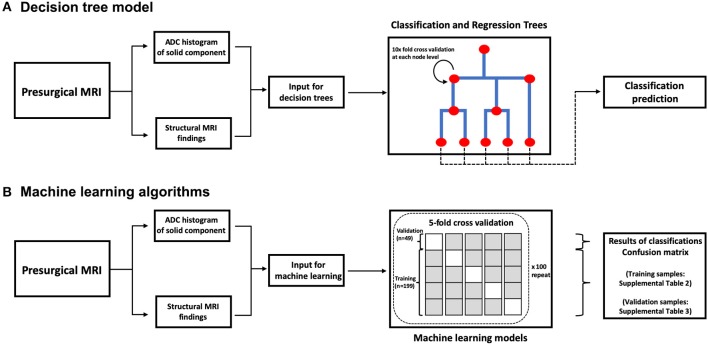
A block diagram of decision tree model **(A)** and machine learning algorithms **(B)**. ADC, apparent diffusion coefficient.

For development of decision trees, we applied the “rpart” R package for Classification and Regression Tree (CART) models (14). At each split/node, a variable is selected to maximize the variance explanation of dependent variable. The patients' characteristics (age and gender), structural MRI findings (Table 2), and ADC histogram metrics were included as input for the model. By default, a 10-fold cross-validation and fitting at each sub-tree were applied. The final classification of the decision tree model was separately compared with the top differential diagnosis from the clinical report and two independent neuroradiologists using the receiver operating characteristics (ROC) analysis implemented by “pROC” package in R (Table 3). We also determined the Cohen's Kappa inter-rater agreement coefficient (Table 4). The CART models were first applied for differentiation of all tumors, and then separately for dichotomized classification of the 5 most common posterior fossa tumors. The block diagram in Figure 2 summarizes the analysis steps in decision tree and machine learning models.

**Table 3:** Comparing the accuracy of decision tree model (Figure 5) with the official clinical interpretation and independent neuroradiologist reviewers.

**Decision tree model**		**Clinical report**		**Reader #1**		**Reader #2**	
	**ROC AUC (95% CI)**	**ROC AUC (95% CI)**	***P-*value**	**ROC AUC (95% CI)**	***P*-value**	**ROC AUC (95% CI)**	***P*-value**
Metastasis	0.857 (0.810–0.904)	0.958 (0.935–0.982)	**0.001**	0.917 (0.877–0.957)	0.056	0.956 (0.935–0.975)	**0.001**
Hemangioblastoma	0.885 (0.826–0.944)	0.891 (0.829–0.951)	0.9148	0.913 (0.858–0.968)	0.510	0.847 (0.779–0.916)	0.448
Pilocytic astrocytoma	0.885 (0.825–0.946)	0.792 (0.718–0.867)	**0.020**	0.855 (0.788–0.922)	0.403	0.830 (0.759–0.901)	0.151
Ependymoma	0.759 (0.663–0.855)	0.857 (0.780–0.934)	0.0841	0.773 (0.678–0.867)	0.838	0.876 (0.801–0.953)	**0.018**
Medulloblastoma	0.8545 (0.767–0.942)	0.788 (0.692–0.884)	0.345	0.856 (0.769_0.944)	0.969	0.912 (0.841–0.983)	0.178
Low grade glioma/astrocytoma	0.6358 (0.515–0.785)	0.721 (0.557–0.885)	0.392	0.737 (0.574–0.901)	0.308	0.815 (0.664–0.965)	0.1049
Atypical teratoid/rhabdoid tumor	0.913 (0.749–1.000)	0.579 (0.415–0.742)	**0.001**	0.742 (0.522–0.961)	0.306	0.750 (0.531–0.969)	0.329

*The receiver operating characteristics (ROC) area under the curve (AUC) with 95% confidence interval (CI) were calculated for the decision tree model (Figure 5) vs. official clinical interpretation, and independent neuroradiologists (separately) in differentiation of posterior fossa tumors. A p-value < 0.05 was considered statistically significant, and depicted in bold*.

**Table 4:** Inter-rater agreement in differentiation of posterior cranial fossa neoplasms.

**Diagnosis**	**Cohen's Kappa**		
	**Clinical report vs Reader #1**	**Clinical report vs Reader #2**	**Reader #1 vs Reader #2**
Metastasis	0.802	0.839	0.787
Hemangioblastoma	0.788	0.820	0.728
Pilocytic astrocytoma	0.749	0.662	0.679
Ependymoma	0.664	0.781	0.677
Medulloblastoma	0.724	0.615	0.823
Low grade glioma/astrocytoma	0.718	0.569	0.423
Lymphoma	0.388	−0.006	−0.006
Anaplastic astrocytoma	0.435	−0.006	−0.006
Atypical teratoid/rhabdoid tumor	0.057	−0.012	0.593
Glioblastoma multiforme	0.559	0.535	0.291
Subependymoma	0.000	0.000	0.063
Choroid plexus papilloma	−0.008	−0.005	−0.005
Ganglioglioma	0.000	0.000	0.000
Rosette-forming glioneuronal tumor	0.000	0.000	0.000

*The Cohen's Kappa co-efficient for inter-rater agreement in classification of each neoplasm type among the official clinical report, and two independent neuroradiologist reviewers*.

### Machine Learning Classification

Different machine learning models were applied and compared for dichotomized classification of the 5 most common posterior fossa tumors—including the naïve Bayes, random forest, support vector machine (SVM), and neural networks (Figure 2). For development of the naïve Bayes models, we applied the “naivebayes” R package with a Laplace smoothing value of 0, as suggested by the package developers. The “randomForest” package was used for random forest ensemble learning classification (15). For each random forest model, 500 trees were constructed applying a randomly-selected one-third of variables at each split (16–19). In our preliminary experiments, the error rate consistently plateaued after constructing 160–300 tree splits in random forests; thus, the default recommendation of 500 trees by the R package deemed adequate to achieve optimal accuracy in our models. For SVM algorithms, we used the “e1071” R package to construct dichotomized classification models. We applied both linear and non-linear kernels for data classification—in this series, polynomial kernel was used for non-linear kernel function. During finetuning of the hyperparameters for SVM models, a cost of 0.1 yielded the optimal error rate, and was applied for all linear kernels. For polynomial kernel, we used a sigma of 1 as the optimal cutoff. For neural networks, we applied the “neuralnet” package, and used the “rBayesianOptimization” package to optimize the number of nodes in the neural network hidden layer.

In order to present realistic estimates from our cohort and minimize the risk of overfitting, we opted to report the averaged results from “stratified” cross-validation. Given the uneven distribution of tumor types in our cohort, a “stratified” sampling strategy seemed appropriate to ensure that enough number of each tumor type is allocated in every training and validation sample. Using stratified random sampling, we applied 5-fold cross validation, preserving the tumor subtype percentage in both training and validation samples. The random sampling was repeated 100 times, and the averaged results from “stratified” cross validation across 500 permutations are presented. In each pair of randomly selected training/validation samples, the model was constructed on training sample, and tested on corresponding validation sample. A confusion matrix was constructed based on prediction results in each training and validation sample, and the corresponding accuracy (number of correctly classified subjects divided by sample size), sensitivity, specificity, positive predictive value (PPV), and negative predictive value (NPV) were calculated. In addition, the ROC area under the curve (AUC) and 95% confidence interval (CI) were computed using 2000 stratified bootstrap replicates per the default implementation in pROC package. The average test characteristics across 500 training and validation samples are reported.

All models were first applied and compared for differentiation of the five most common posterior fossa tumors—with each combination of tumor type and machine learning analyzed separately. Given that random forest models yielded higher accuracy for classification of tumor types compared to other algorithms, we applied the random forest for multi-class differentiation of all tumor types. The average multiclass ROC AUC was determined for random forest models and neuroradiologist interpretations using the “multiROC” package. Notably, comparison and calculation of 95% CI for multiclass averaged AUC is not feasible. In addition, the averaged “mean decrease in Gini coefficient” are reported to depict the relative effect of each variable on random forest model accuracy if the variable is deleted.

### Statistical Analysis

The data are expressed as mean ± standard deviation, and frequency (percentage). Kolmogorov–Smirnov test confirmed normal distribution of continuous variables in our analysis. For univariate comparison between different tumor types, the ANOVA with Tukey *post-hoc* analysis was used for continuous variables, and Chi square test was used for nominal variables. MANOVA was applied to evaluate the effects of 1.5 vs. 3 Tesla scanners on ADC measurement. In addition to R package (https://cran.r-project.org/), we used SPSS 22.0 (IBM, Somers, NY) for statistical analysis.

## Results

### Posterior Fossa Tumors

Of 403 consecutive patients with pathologic diagnosis of posterior cranial fossa neoplasm over 12-year period, 256 patients had intra-axial/intra-ventricular tumors. We excluded 136 patients with extra-axial tumors (except intraventricular tumors), and 11 subjects with poor quality of MRI. Excluded extra-axial tumors were schwannoma, meningioma, metastases, and hemangiopericytoma. Among tumors included in our analysis (Table 1), metastasis, hemangioblastoma, pilocytic astrocytoma, ependymoma, and medulloblastoma were the most common types, comprising 205/256 (80%) subjects. Representative tumors from different pathologies are depicted in Figure 1.

### Patients' Characteristics and Qualitative MRI Analysis

**Figure 3:**
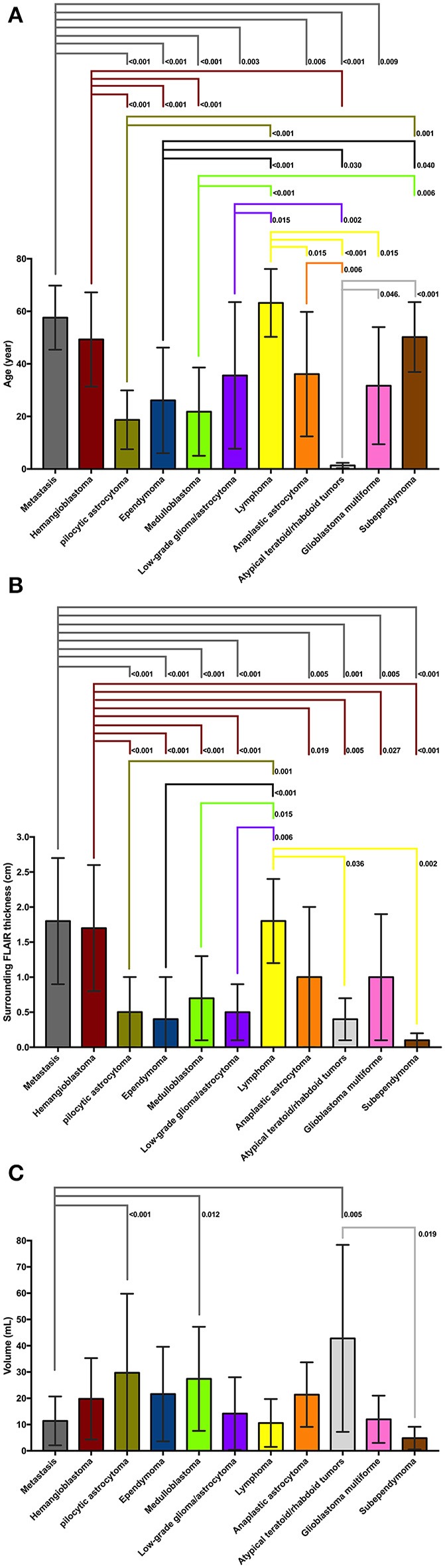
**(A)** On *post-hoc* analysis, patients with atypical teratoid/rhabdoid tumors (ATRT) had significantly lower age at presentation compare to all other tumor types except medulloblastoma and pilocytic astrocytoma. Patients with medulloblastoma, pilocytic astrocytoma, and ependymoma were significantly younger compared to those with metastasis, lymphoma, hemangioblastoma, and subependymoma. Patients with metastasis and hemangioblastomas were also older than those with anaplastic astrocytoma, low-grade glioma, and glioblastoma multiforme. **(B)** On *post hoc* analysis, metastases, hemangioblastomas, and lymphomas had larger diameter of peritumoral FLAIR hyperintensity compared to medulloblastoma, pilocytic astrocytomas, ependymomas, low-grade glioma, ATRT, and subependymomas—likely since latter tumors tend to be intraventricular with virtually no peritumoral edema. Also, the peritumoral FLAIR hyperintensity surrounding metastases, and hemangioblastomas was larger in diameter compared to anaplastic astrocytoma, and glioblastoma multiforme. **(C)** On *post hoc* analysis of tumor volumes, pilocytic astrocytomas, medulloblastomas, and ATRTs had larger size compared to metastases. The ATRTs were also significantly larger compared to subependymomas. ATRT, atypical teratoid/rhabdoid tumors; FLAIR, fluid attenuated inversion recovery.

A summary of the univariate analysis comparing various posterior fossa tumors is shown in Table 2. The patients' age at presentation, tumor lesion localization, tumor morphology, enhancement pattern, degree of peritumoral FLAIR hyperintensity, whole tumor volume, and presence of hydrocephalus were significantly different among tumor types in univariate analyses. The results of *post-hoc* analysis for patients' age, peri-tumor FLAIR hyperintensity width, and tumor volume between different neoplasms are depicted in Figure 3.

### ADC Histogram Analysis

**Figure 4:**
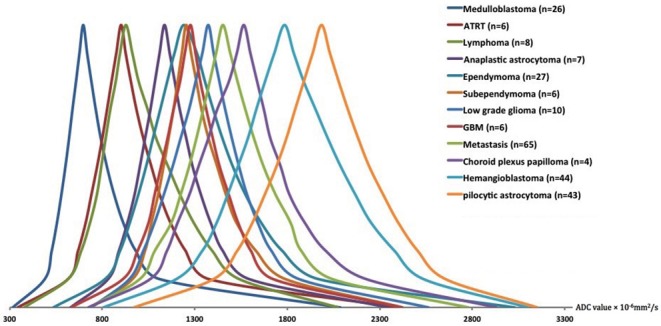
The schematic representation of the averaged ADC histogram distribution among different posterior fossa neoplasms. Medulloblastomas, ATRT, and lymphomas had the lowest; whereas, pilocytic astrocytomas, and hemangioblastomas had the highest ADC histogram percentile values. The average percentile values for each tumor type were calculated, and representative averaged histograms were modified so that the median values would be depicted at the same height on the y axis. ADC, apparent diffusion coefficient; ATRT, atypical teratoid/rhabdoid tumors; GBM, glioblastoma multiforme.

Figure 4 depicts the schematic representation of the averaged ADC percentile values among different posterior fossa tumors. Medulloblastomas, followed by ATRT and lymphomas had the lowest ADC histogram percentile values; whereas, pilocytic astrocytomas, followed by hemangioblastomas had the highest ADC histogram percentile values (Figure 4). Using ANOVA, there was significant difference in all ADC percentile metrics, average, skewness, and kurtosis values among 11 different tumor types (with ≥6 subjects) in our cohort (*p*-values < 0.001). There has been no significant difference in ADC histogram metrics between DWI series from 1.5 Tesla (*n* = 34) vs. 3 Tesla (*n* = 214) scanners in MANOVA.

### Decision Tree Models for Differentiation of Posterior Fossa Tumors

**Figure 5:**
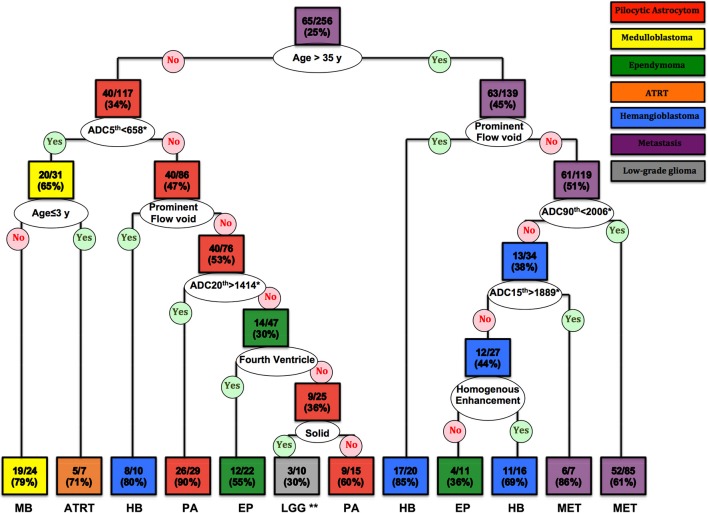
The CART decision tree model could differentiate 7 histopathologies among posterior fossa tumors. In each box (node/leaf), the accurate classification rate (positive predictive value) is expressed as ratios of the most common tumor type per total number of cases fulfilling the criteria. Each box is colored to represent the tumor type, and the intensity of the color reflects the accurate classification ratio. ADC, apparent diffusion coefficient; ATRT, atypical teratoid/rhabdoid tumors; CART, Classification and Regression Tree; EP, Ependymoma; HB, Hemangioblastoma; LGG, low-grade glioma/astrocytoma; MET, metastases; MB, Medulloblastoma; PA, pilocytic astrocytoma. *The ADC n^th^ refers to the ADC histogram n^th^ percentile value ×10^−6^ mm^2^/s; for example, ADC 5th percentile <658 × 10^−6^ mm^2^/s. **Both low-grade glioma and anaplastic astrocytoma had 3/10 (30%) ratios in this terminal node.

The CART decision tree model successfully differentiated 7 types of neoplasms in our cohort (Figure 5). The first decision node identified by the model was patients' age with a cut off of 35 years. Subsequent nodes used ADC histogram values, presence/absence of prominent flow voids on T2 weighted images, homogenous enhancement pattern, solid tumor morphology, and the fourth ventricle localization, respectively, for further tumor classification (Figure 5). The terminal nodes (leaves) of the decision tree yielded 30 to 90% correct classification ratios. Moreover, the likelihood of each tumor type, based on classification criteria is calculated in each terminal node (Figure 5).

Using ROC curve analysis, we compared the accuracy of the decision tree model with clinical interpretation, and each of independent neuroradiologists (Table 3). The decision tree model yielded a greater AUC compared to the clinical interpretation in differentiation of the pilocytic astrocytoma (*p* = 0.020) and ATRT (*p* = 0.001) from other neoplasm subtypes; whereas, the clinical interpretation and reviewer #2 had higher ROC AUC in differentiation of metastasis (*p* = 0.001) from other tumors. The Cohen's Kappa analysis, showed substantial inter-rater agreement (>0.6) between the clinical interpretation and neuroradiologists among the 5 most common tumor types; however, the agreement rates were lower for the less common tumors (Table 4).

**Figure 6:**
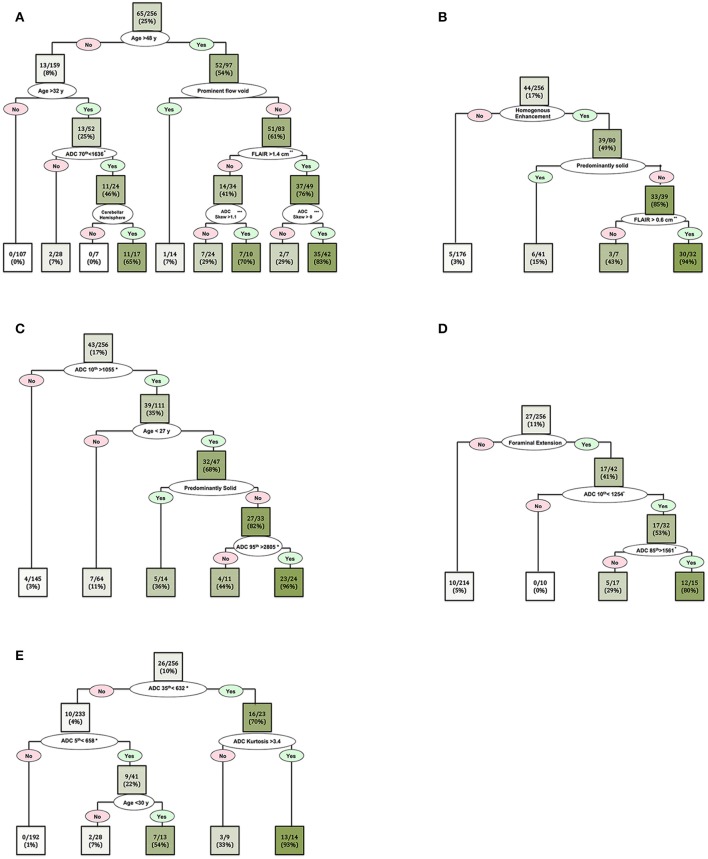
Decision tree models for dichotomized differentiation of the 5 most common posterior cranial fossa neoplasms: **(A)** metastasis, **(B)** hemangioblastomas, **(C)** pilocytic astrocytoma, **(D)** ependymomas, and **(E)** medulloblastomas. In each box (node/leaf), the accurate classification rate (positive predictive value) is expressed as ratios of the most common tumor type per total number of cases fulfilling the criteria. The intensity of the green color reflects the accurate classification ratio. Froaminal extension refers to tumoral extension through the foramina of Luschka and/or Magendie. *The ADC n^th^ refers to the ADC histogram n^th^ percentile value ×10^−6^ mm^2^/s; for example, ADC 70th percentile <1,636 × 10^−6^ mm^2^/s. **Peritumoral FLAIR hyperintensity width. ***ADC histogram skewness.

In order to further delineate specific imaging characteristics of common posterior fossa tumors and achieve higher classification accuracy, we also developed separate CART decision trees models for dichotomized classification of the 5 most common tumors in our cohort (Figure 6). The patients' age, ADC histogram metrics, peritumoral FLAIR hyperintensity width, presence of prominent flow void, enhancement pattern, presence of cystic component, fourth ventricle location, cerebellar hemisphere localization, extension through foramina of Luschka/Magendie, and tumor volume, were included in these CART decision tree models (Figure 6).

### Machine Learning Algorithm for Tumor Classification

**Figure 7:**
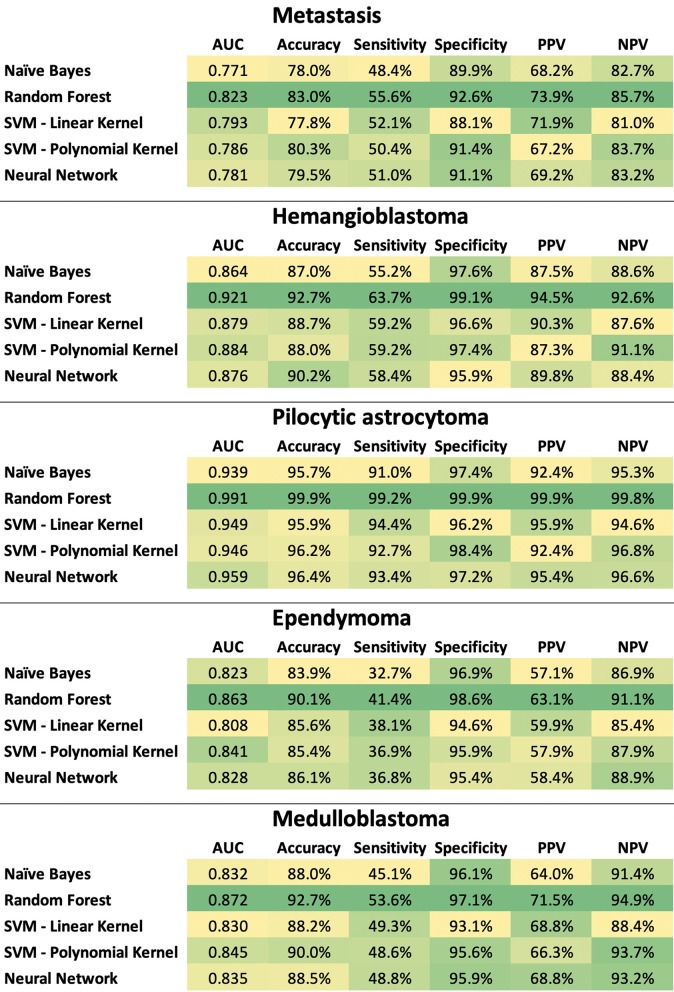
Heat map summary for classification performance of different machine learning algorithms in dichotomized differentiation of the 5 most common posterior fossa tumors. The test characteristics were calculated in validation datasets from ×100 repeats of 5-fold cross validation– details in Supplemental Tables 2, 3. NPV, negative predictive value; PPV, positive predictive value; SVM, support vector machine.

The ratio of tumor types included in the stratified training (*n* = 199) and validation (*n* = 49) datasets are tabulated in Supplemental Table 1. In separate classification models devised for the dichotomized differentiation of the 5 most common posterior fossa tumors, random forest models achieved the highest ROC AUC, sensitivity, specificity, PPV, and NPV across training and validation samples from the ×100 repeat of 5-fold stratified cross validation (Figure 7, Supplemental Tables 2, 3).

**Figure 8:**
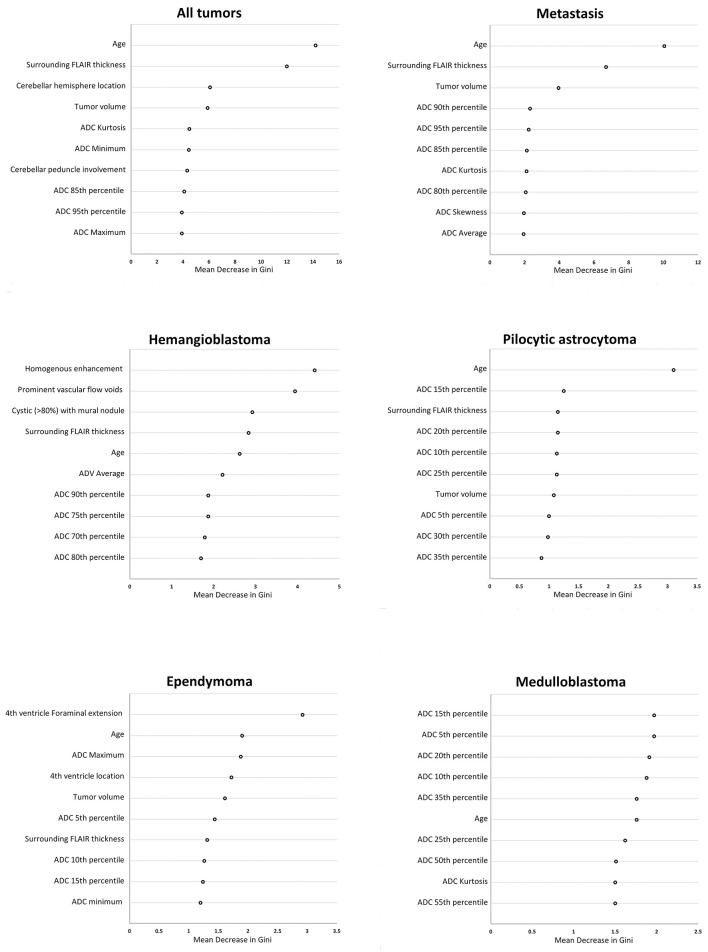
The “mean decrease in Gini coefficient” in random forest models for differentiation of posterior cranial fossa tumors. Separate random forests models were developed for differentiation of all posterior fossa tumors from each other in multiclass analysis, as well as dichotomized classification for the 5 most common posterior fossa neoplasms. The top 10 variables with the highest averaged “mean decrease in Gini coefficient” among from ×100 repeats of 5-fold cross validation are reported. ADC, apparent diffusion coefficient; FLAIR, fluid attenuated inversion recovery.

Then, we applied random forest model for multiclass differentiation of posterior fossa tumor types. Using a multiclass ROC analysis, the average AUC of random forest models was 0.961 in training datasets, and 0.873 in validation dataset. Using multiclass ROC analysis in same 248 patients, the average AUC of clinical interpretation, reviewer #1, and reviewer #2 were 0.832, 0.799, and 0.834, respectively. There was significant correlation between pathological diagnosis and random forest model prediction in the training (averaged *r* = 0.96, *p* < 0.001), and validation (averaged *r* = 0.51, *p* < 0.001) datasets. The patients' age, width of peritumoral FLAIR hyperintensity, cerebellar hemisphere location, involvement of cerebellar peduncle, tumor volume, and ADC histogram metrics had the greatest impact on accuracy of random forest models (Figure 8).

## Discussion

Using the CART decision tree model analysis, we have devised differentiation algorithms for posterior fossa tumors based on patients' age, ADC histogram analysis, and qualitative imaging features on pretreatment MRI. The proposed decision tree model (in Figure 5) could differentiate 7 histopathologies with 30 to 90 % accurate classification rates in terminal nodes. This decision tree model appears to be most helpful in differentiation of pilocytic astrocytoma and ATRT - as it achieved higher accuracy compared to clinical report in our cohort. We also demonstrated the feasibility of random forest machine learning algorithms in devising classification models for differentiation of posterior fossa tumors. Applying multiclass ROC analysis, we achieved an averaged AUC of 0.961 in training datasets, and 0.873 in validation dataset, as compared to 0.799 and 0.834 by neuroradiologists.

Recent studies demonstrated the feasibility of machine learning algorithms in prediction of glioma histopathological grade, and classification of the most common pediatric posterior fossa tumors (20–22). These studies, however, utilized small training datasets with few select types of tumors, therefore clinical application of these models may be limited (23). In our study, we used a large comprehensive cohort of patients including 15 different types of posterior fossa tumors and at least 6 representative patients for each tumor histology. Our decision tree models rely on qualitative imaging features and quantitative histogram analysis, which can be easily translated to commercially-available image viewer systems in clinical practice; thus, providing a ready-to-apply tool for neuroradiologists to formulate their differential diagnosis before pathology results becomes available. The decision tree model and the “mean decrease in Gini coefficient” in random forest models also provide an insight into the innerworkings of machine learning models in their prediction decision.

The patients' age is one of the most important factors in differentiation of posterior fossa tumors, and it is well established that adult and pediatric patients are prone to different types of posterior fossa tumors. In this study, instead of using preset age cutoffs as inclusion or exclusion criteria, we applied CART models to identify data driven and tumor-specific age thresholds for differentiation of various neoplasms; and indeed, an age cutoff of 35 years was the first step in decision tree model for differentiation of various tumor types (Figure 5). Moreover, in our cohort, an age cutoff of ≤ 3 years was identified as differentiation criteria for ATRT from other tumors—including medulloblastoma (Figure 5); and an age cutoff of <27 years was helpful for differentiation of pilocytic astrocytoma from rest of tumors (Figure 6C).

While similar qualitative and quantitative imaging characteristics were previously used to differentiate posterior fossa tumors, our decision tree model provides step-wise approach for differentiation of wide various posterior fossa tumors (Figures 5, 6, 8). For example, high ADC values in solid component of the tumor and young age at presentation could help differentiate pilocytic astrocytoma from other tumors in posterior fossa (Figures 5, 6C). In dichotomized analysis, age younger than 27 years, high ADC percentile values (10th percentile >1,055 × 10^−6^ mm^2^/s and 95th percentile >2,805 × 10^−6^ mm^2^/s), and presence of cystic component had 96% accurate classification rate (positive predictive value) for differentiation of pilocytic astrocytoma from other tumors (Figure 6C). On the other hand, age at presentation of <35 year and low ADC values are suggestive of medulloblastoma (or ATRT), regardless of extension through foramina of Luschka/Magendie, tumor localization, or enhancement pattern (Figures 5, 6E). Although the number of ATRT patients in our cohort was too small to draw a firm conclusion, we found that an age cutoff ≤ 3 years can help differentiate ATRT from other neoplasms of posterior fossa—including medulloblastoma (Figure 5). Indeed, an ADC 5th percentile <658 × 10^−6^ mm^2^/s and age ≤ 3 years of age had 71% accuracy (positive predictive value) for differentiation of ATRT from other posterior fossa tumors (Figure 5).

In this study, we included consecutive patients with posterior cranial fossa tumors, which is a distinction from many prior studies restricting their cohorts based on age, select tumor types, or location (5, 8, 11, 12, 24). In terms of image analysis, prior research focused on 2D region of interest measurements, restricted use of ADC histogram metrics, and exclusion of qualitative features from analysis (6, 8, 11, 12). The strength of our analysis is defined by volumetric voxel-based ADC histogram analysis, use of comprehensive ADC histogram metrics, and incorporation of qualitative imaging analysis and patient's age (6, 8, 11, 12).

Almost all prior machine learning schemes for differentiation of posterior fossa tumors have limited their study to differentiation of ependymoma, medulloblastoma, and pilocytic astrocytoma (25). Rodriguez Gutierrez et al. have applied support vector machine classification of 17 medulloblastomas, 16 pilocytic astrocytomas, and 7 ependymomas (8). They reported that combination of the ADC histogram 25th percentile, 75th percentile, and skewness values could achieve the highest accuracy of 91% (8). Orphanidou-Vlachou et al. have applied principal component analysis for feature selection in combination with probabilistic neural network to classify 21 medulloblastomas, 14 pilocytic astrocytomas and 5 ependymomas patients based on T1- and T2-weighted image texture features (8, 25). In leave-one-out cross validation, they achieved 85.8% overall accuracy (25). Fetit et al. analyzed 21 medulloblastomas, 20 pilocytic astrocytomas and 7 ependymomas patients; and compared naïve Bayes, classification tree, k nearest neighbor, SVM, artificial neural network, and logistic regression classification models using three-dimensional texture data (26). In leave-one-out cross-validation, the SVM and artificial neural networks achieved the highest accuracy of 92% (26). In 2017, Zarinabad et al. reported the results of 1.5 Tesla 1H-MR spectroscopy for differentiation of 42 pilocytic astrocytoma, 38 medulloblastomas, and 10 ependymomas, comparing Naïve Bayes, SVM, artificial neural networks, and linear discriminative analysis (27). Using AdaBoost ensemble technique and synthetic minority oversampling technique (SMOTE), they could achieve an averaged balanced accuracy rate of 91% in oversampled-data based on metabolite concentration (27). In 2018, Zarinabad et al. reported the results of 3 Tesla 1H-MR spectroscopy for differentiation of 17 medulloblastomas, 20 pilocytic astrocytomas, and 4 ependymomas, and could achieve the highest Balanced Accuracy Rate of 86% using SVM classifiers (28). In our study, there was no exclusion based on the patients' age or the tumor histopathology, and we could achieve 0.873 averaged AUC among validation datasets for differentiation of 11 posterior fossa tumor types in the multiclass random forest analysis.

Of note, ADC histogram metrics were among variables with the greatest effects on accuracy of random forest models for differentiation of posterior fossa tumors (Figure 8). While prior studies have shown the value of ADC maps in differentiation of posterior fossa tumors (8–10), current results depict how combination of ADC histogram analysis and qualitative MR imaging features defined by neuroradiologists can help with diagnostic differentiation of these tumors. For example, among adult posterior fossa tumors, both metastases and hemangioblastomas present with prominent surrounding vasogenic edema (Figure 3). However, homogenous enhancement pattern, presence of prominent vascular flow voids, and higher ADC histogram percentile values favor the diagnosis of hemangioblastoma over metastasis (Figures 5, 6).

While individual CART decision trees are prone to overfitting, random forest ensemble learning method theoretically reduces the potential overfitting. In addition, we opted to report the averaged results of machine learning models among 500 randomly selected training and validation cohorts to represent a realistic reflection of machine learning algorithm accuracy for prediction of tumor type, and compensate for potential overfitting. By doing so, however, we could not directly compare the performance of machine learning algorithm with clinical interpretation or neuroradiologist results. Nevertheless, the results of multiclass ROC analysis as well as the ROC AUC of reviewers in Table 3 and averaged ROC AUC of machine learning models can provide an indirect comparison between neuroradiologist interpretation and machine learning models.

One of the strengths of our study is the use of a large cohort of patients presenting with posterior fossa tumor, which is representative of a patient population in a tertiary care center. A large and diverse cohort allowed us to have an appropriate training set for development of an accurate machine learning based model that differentiates a wide variety of posterior fossa tumors encountered in a tertiary care center practice. The natural next step of current study is training of machine learning models for prediction of molecular subtypes in specific posterior tumors. Future studies and prospective validation of decision tree models can also determine the impact of proposed machine learning algorithms on pretreatment diagnosis and therapy strategies in patients with posterior fossa tumors. Our results, however, provide the first step in devising a “no priori” and “data driven” decision models for differentiation of posterior fossa tumors, and are a new guide for methodological design of future machine learning classifiers. In addition, combination of clinical, and genetic biomarkers with imaging features can provide multivariate wholistic models for accurate prognostication and targeted therapy plan.

The major limitations of current study are the small number of rare tumor types; and the lack of molecular subtyping in medulloblastomas and ependymomas, which affect neoplasm prognosis and treatment planning (29). The study is also inherently limited in devising statistically powerful diagnostic models for less frequent posterior fossa tumors. Moreover, we only included subjects with known posterior fossa tumor; whereas, the machine learning model should preferably differentiate non-neoplastic tumor-mimics from tumors. However, designing the selection criteria for inclusion of potential tumor-mimic lesions for training machine learning models can be challenging due to lack of consensus on which lesions are qualified as tumor-mimic. Manual segmentation of brain tumors and measurement of peritumoral FLAIR hyperintensity can be challenging and a source of variability, particularly in non-enhancing T2 hyperintense glial tumors. Acquisition of MRIs in two different field strengths and on various scanners may also introduce heterogeneity in our data, although ADC obtained with repetition time >3,000 ms and b value of 1,000 s/mm^2^ are not substantially affected by scanner magnet strength (30). Additionally, there was no homogenous standardized imaging performed. Finally, the difference in imaging protocols, heterogeneity of patients' population, and age group can limit generalizability of our models.

## Conclusion

We developed objective and quantitative decision tree models for differentiation of posterior fossa tumors based on ADC histogram metrics, patients' age, and qualitative MR imaging features that can easily be extracted on common image viewer platforms by radiologists. In addition, we have compared different machine learning classifiers for prediction of the most common posterior fossa tumors, and found that random forest models achieved greater accuracy in tumor differentiation. However, the results of our study need to be used with caution; and the proposed differentiation model should be validated in a larger prospective cohort before being used for clinical decision making. Pending prospective validation, such quantitative and objective diagnostic tools can potentially guide surgical planning or treatment decision for presurgical neoadjuvant therapy.

## Data Availability Statement

The datasets generated for this study are available on request to the corresponding author.

## Ethics Statement

The studies involving human participants were reviewed and approved by Institutional Review Board at UCSF. Written consent requirement was waved given the retrospective review nature of the study.

## Author Contributions

SP and SC: guarantors of integrity of entire study, concepts and design, and data acquisition or data analysis/interpretation. All authors: manuscript drafting and final version approval.

### Conflict of Interest

The authors declare that the research was conducted in the absence of any commercial or financial relationships that could be construed as a potential conflict of interest.
