# Supplementary material for: Machine Learning Decision Tree Models for Differentiation of Posterior Fossa Tumors Using Diffusion Histogram Analysis and Structural MRI Findings
Source: Front Oncol. 2020 Feb 7;10:71. doi: 10.3389/fonc.2020.00071 (PMC7018938; doi:10.3389/fonc.2020.00071)
Supplement: Supplementary file 1 [file Table_1.docx]

**Supplemental online Table 1**. Stratified distribution of cranial fossa tumors among the training and validation datasets for machine learning models for cross validation.

| **Diagnosis** | **Training dataset**  (N=199) | **Validation dataset**  (N=49) |
| --- | --- | --- |
| **Metastasis** | 52 (26.1%) | 13 (26.5%) |
| **Hemangioblastoma** | 35 (17.6%) | 9 (18.4%) |
| **Pilocytic astrocytoma** | 34 (17.1%) | 9 (18.4%) |
| **Ependymoma** | 22 (11.1%) | 5 (10.2%) |
| **Medulloblastoma** | 21 (10.6%) | 5 (10.2%) |
| **Low grade glioma/astrocytoma** | 8 (4%) | 2 (4.1%) |
| **Lymphoma** | 6 (3%) | 2 (4.1%) |
| **Anaplastic astrocytoma** | 6 (3%) | 1 (2%) |
| **Atypical teratoid/rhabdoid tumor** | 5 (2.5%) | 1 (2%) |
| **Glioblastoma multiforme** | 5 (2.5%) | 1 (2%) |
| **Subependymoma** | 5 (2.5%) | 1 (2%) |
