# Supplementary material for: Machine Learning Decision Tree Models for Differentiation of Posterior Fossa Tumors Using Diffusion Histogram Analysis and Structural MRI Findings
Source: Front Oncol. 2020 Feb 7;10:71. doi: 10.3389/fonc.2020.00071 (PMC7018938; doi:10.3389/fonc.2020.00071)
Supplement: Supplementary file 2 [file Table_2.docx]

**Supplemental table 2**. Comparative test characteristics for different machine learning algorithms among training datasets.

| **Metastasis** | | | | | |
| --- | --- | --- | --- | --- | --- |
|  | **Accuracy** | **Sensitivity** | **Specificity** | **PPV** | **NPV** |
| **Naive Bayes** | 0.861  (0.841-0.881) | 83.0%  (79.0%-87.0%) | 58.4%  (54.9%-61.9%) | 96.9%  (93.9%-99.9%) | 74.2%  (70.2%-78.2%) |
| **Random forest** | 0.923  (0.893-0.953) | 90.0%  (86.5%-93.5%) | 61.6%  (58.1%-65.1%) | 97.6%  (95.6%-99.6%) | 78.9%  (76.9%-80.9%) |
| **SVM – linear kernel** | 0.903  (0.868-0.938) | 83.8%  (80.3%-87.3%) | 63.1%  (59.6%-66.6%) | 97.1%  (93.6%-100.0%) | 82.9%  (79.4%-86.4%) |
| **SVM – polynomial kernel** | 0.866  (0.831-0.901) | 88.3%  (85.8%-90.8%) | 60.4%  (57.4%-63.4%) | 97.4%  (94.4%-100.0%) | 78.2%  (75.2%-81.2%) |
| **Neural Network** | 0.841  (0.801-0.881) | 89.5%  (87.0%-92.0%) | 56.0%  (52.5%-59.5%) | 98.1%  (96.1%-100.0%) | 75.2%  (72.7%-77.7%) |
| **Hemangioblastoma** | | | | | |
|  | **Accuracy** | **Sensitivity** | **Specificity** | **PPV** | **NPV** |
| **Naive Bayes** | 0.934 (  0.904-0.964) | 95.0%  (91.5%-98.5%) | 61.2%  (57.7%-64.7%) | 99.9%  (97.4%-100.0%) | 92.5%  (89.0%-96.0%) |
| **Random forest** | 0.971  (0.931-1.000) | 99.9%  (97.4%-100.0%) | 72.7%  (70.7%-74.7%) | 99.9%  (96.9%-100.0%) | 99.9%  (96.9%-100.0%) |
| **SVM – linear kernel** | 0.959  (0.929-0.989) | 98.7%  (95.2%-100.0%) | 70.2%  (68.2%-72.2%) | 99.9%  (95.9%-100.0%) | 99.3%  (95.8%-100.0%) |
| **SVM – polynomial kernel** | 0.994  (0.959-1.000) | 94.0%  (91.5%-96.5%) | 66.2%  (62.7%-69.7%) | 99.9%  (97.9%-100.0%) | 95.3%  (92.3%-98.3%) |
| **Neural Network** | 0.966  (0.936-0.996) | 96.2%  (92.2%-100.0%) | 68.4%  (65.4%-71.4%) | 99.9%  (97.4%-100.0%) | 97.8%  (95.3%-100.0%) |
| **Pilocytic astrocytoma** | | | | | |
|  | **Accuracy** | **Sensitivity** | **Specificity** | **PPV** | **NPV** |
| **Naive Bayes** | 0.999  (0.979-1.000) | 99.9%  (95.9%-100.0%) | 98.0%  (96.0%-100.0%) | 99.9%  (95.9%-100.0%) | 99.9%  (96.4%-100.0%) |
| **Random forest** | 0.999  (0.974-1.000) | 99.9%  (95.9%-100.0%) | 99.9%  (97.9%-100.0%) | 99.9%  (96.4%-100.0%) | 99.9%  (97.9%-100.0%) |
| **SVM – linear kernel** | 0.999  (0.974-1.000) | 99.9%  (96.4%-100.0%) | 99.9%  (96.4%-100.0%) | 99.9%  (96.4%-100.0%) | 99.9%  (96.9%-100.0%) |
| **SVM – polynomial kernel** | 0.999  (0.979-1.000) | 99.9%  (96.9%-100.0%) | 99.9%  (97.4%-100.0%) | 99.9%  (97.4%-100.0%) | 98.4%  (96.4%-100.0%) |
| **Neural Network** | 0.999  (0.964-1.000) | 99.9%  (97.4%-100.0%) | 99.9%  (96.4%-100.0%) | 99.9%  (97.4%-100.0%) | 99.9%  (96.4%-100.0%) |
| **Ependymoma** | | | | | |
|  | **Accuracy** | **Sensitivity** | **Specificity** | **PPV** | **NPV** |
| **Naive Bayes** | 0.913  (0.873-0.953) | 93.9%  (90.4%-97.4%) | 37.7%  (33.7%-41.7%) | 99.9%  (97.4%-100.0%) | 68.1%  (66.1%-70.1%) |
| **Random forest** | 0.923  (0.883-0.963) | 96.1%  (93.6%-98.6%) | 46.4%  (43.9%-48.9%) | 99.9%  (96.9%-100.0%) | 74.1%  (70.1%-78.1%) |
| **SVM – linear kernel** | 0.918  (0.878-0.958) | 92.6%  (89.6%-95.6%) | 43.1%  (39.1%-47.1%) | 99.9%  (97.9%-100.0%) | 66.9%  (62.9%-70.9%) |
| **SVM – polynomial kernel** | 0.901  (0.861-0.941) | 90.4%  (87.4%-93.4%) | 43.9%  (40.4%-47.4%) | 99.9%  (96.4%-100.0%) | 67.9%  (64.9%-70.9%) |
| **Neural Network** | 0.928  (0.908-0.948) | 91.1%  (87.1%-95.1%) | 46.8%  (43.8%-49.8%) | 99.9%  (96.9%-100.0%) | 64.4%  (61.4%-67.4%) |
| **Medulloblastoma** | | | | | |
|  | **Accuracy** | **Sensitivity** | **Specificity** | **PPV** | **NPV** |
| **Naive Bayes** | 0.892  (0.867-0.917) | 96.0%  (93.5%-98.5%) | 56.1%  (53.1%-59.1%) | 99.9%  (95.9%-100.0%) | 74.0%  (70.5%-77.5%) |
| **Random forest** | 0.982  (0.962-1.000) | 99.9%  (96.9%-100.0%) | 62.6%  (59.6%-65.6%) | 99.9%  (95.9%-100.0%) | 80.5%  (77.0%-84.0%) |
| **SVM – linear kernel** | 0.920  (0.895-0.945) | 95.2%  (91.7%-98.7%) | 54.3%  (50.3%-58.3%) | 99.9%  (97.4%-100.0%) | 76.8%  (74.8%-78.8%) |
| **SVM – polynomial kernel** | 0.945  (0.925-0.965) | 99.9%  (96.4%-100.0%) | 56.6%  (53.6%-59.6%) | 99.9%  (97.4%-100.0%) | 72.3%  (69.3%-75.3%) |
| **Neural Network** | 0.885  (0.845-0.925) | 94.5%  (92.0%-97.0%) | 55.8%  (52.8%-58.8%) | 99.9%  (97.9%-100.0%) | 77.8%  (75.8%-79.8%) |

Detailed results for different machine learning algorithms for differentiation of the 5 most common posterior fossa tumors among training datasets. The results are the averaged (95% confidence interval) performance for ×100 repeats 5-fold cross validation (×500 randomly selected training samples).
