# Supplementary material for: Machine Learning Decision Tree Models for Differentiation of Posterior Fossa Tumors Using Diffusion Histogram Analysis and Structural MRI Findings
Source: Front Oncol. 2020 Feb 7;10:71. doi: 10.3389/fonc.2020.00071 (PMC7018938; doi:10.3389/fonc.2020.00071)
Supplement: Supplementary file 3 [file Table_3.docx]

**Supplemental table 3**. Comparative test characteristics for different machine learning algorithms among validation datasets.

| **Metastasis** | | | | | |
| --- | --- | --- | --- | --- | --- |
|  | **Accuracy** | **Sensitivity** | **Specificity** | **PPV** | **NPV** |
| **Naive Bayes** | 0.771  (0.723 - 0.818) | 78.0%  (73.0% - 81.5%) | 48.4%  (41.6% - 51.4%) | 89.9%  (84.4% - 95.1%) | 68.2%  (62.7% - 72.9%) |
| **Random forest** | 0.823  (0.767 - 0.879) | 83.0%  (78.8% - 86.8%) | 55.6%  (50.9% - 61.4%) | 92.6%  (86.1% - 98.4%) | 73.9%  (69.4% - 79.4%) |
| **SVM – linear kernel** | 0.793  (0.743 - 0.836) | 77.8%  (73.8% - 83.0%) | 52.1%  (45.1% - 57.3%) | 88.1%  (85.1% - 95.1%) | 71.9%  (66.4% - 76.4%) |
| **SVM – polynomial kernel** | 0.786  (0.743 - 0.833) | 80.3%  (73.5% - 84.8%) | 50.4%  (45.1% - 55.6%) | 91.4%  (85.1% - 96.4%) | 67.2%  (61.4% - 73.9%) |
| **Neural Network** | 0.781  (0.751 - 0.831) | 79.5%  (75.5% - 84.3%) | 51.0%  (45.3% - 54.8%) | 91.1%  (85.9% - 94.9%) | 69.2%  (65.9% - 75.4%) |
| **Hemangioblastoma** | | | | | |
|  | **Accuracy** | **Sensitivity** | **Specificity** | **PPV** | **NPV** |
| **Naive Bayes** | 0.864  (0.809 - 0.916) | 87.0%  (82.5% - 92.5%) | 55.2%  (51.7% - 61.7%) | 97.6%  (91.9% - 99.9%) | 87.5%  (83.5% - 93.5%) |
| **Random forest** | 0.921  (0.856 - 0.986) | 92.7%  (88.7% - 96.2%) | 63.7%  (59.2% - 68.2%) | 99.1%  (93.4% - 99.9%) | 94.5%  (89.5% - 98.8%) |
| **SVM – linear kernel** | 0.879  (0.836 - 0.931) | 88.7%  (83.0% - 93.7%) | 59.2%  (53.2% - 63.4%) | 96.6%  (91.6% - 99.9%) | 90.3%  (86.0% - 96.8%) |
| **SVM – polynomial kernel** | 0.884  (0.834 - 0.929) | 88.0%  (84.2% - 93.0%) | 59.2%  (53.5% - 63.0%) | 97.4%  (92.1% - 99.9%) | 87.3%  (83.3% - 92.8%) |
| **Neural Network** | 0.876  (0.851 - 0.934) | 90.2%  (85.5% - 93.0%) | 58.4%  (55.4% - 65.4%) | 95.9% (93.1% - 99.9%) | 89.8%  (86.3% - 95.8%) |
| **Pilocytic astrocytoma** | | | | | |
|  | **Accuracy** | **Sensitivity** | **Specificity** | **PPV** | **NPV** |
| **Naive Bayes** | 0.939  (0.879 - 0.979) | 95.7%  (90.9% - 99.9%) | 91.0%  (86.7% - 95.5%) | 97.4%  (91.9% - 99.9%) | 92.4%  (89.4% - 97.7%) |
| **Random forest** | 0.991  (0.931 - 0.999) | 99.9%  (94.2% - 99.9%) | 99.2%  (94.5% - 99.9%) | 99.9%  (95.2% - 99.9%) | 99.9%  (95.9% - 99.9%) |
| **SVM – linear kernel** | 0.949  (0.896 - 0.999) | 95.9%  (89.4% - 99.2%) | 94.4%  (90.7% - 99.9%) | 96.2%  (90.4% - 99.9%) | 95.9%  (90.4% - 99.9%) |
| **SVM – polynomial kernel** | 0.946  (0.899 - 0.999) | 96.2%  (91.2% - 99.9%) | 92.7%  (89.0% - 97.7%) | 98.4%  (92.2% - 99.9%) | 92.4%  (89.7% - 98.7%) |
| **Neural Network** | 0.959  (0.896 - 0.999) | 96.4%  (90.7% - 99.9%) | 93.4%  (88.6% - 98.4%) | 97.2%  (92.7% - 99.9%) | 95.4%  (91.7% - 99.9%) |
| **Ependymoma** | | | | | |
|  | **Accuracy** | **Sensitivity** | **Specificity** | **PPV** | **NPV** |
| **Naive Bayes** | 0.823  (0.753 - 0.856) | 83.9%  (79.6% - 88.9%) | 32.7%  (29.2% - 38.2%) | 96.9%  (91.6% - 99.9%) | 57.1%  (51.6% - 62.6%) |
| **Random forest** | 0.863  (0.793 - 0.933) | 90.1%  (85.9% - 93.6%) | 41.4%  (35.2% - 45.9%) | 98.6%  (93.4% - 99.9%) | 63.1%  (57.9% - 66.6%) |
| **SVM – linear kernel** | 0.808  (0.783 - 0.868) | 85.6%  (79.4% - 89.9%) | 38.1%  (31.1% - 42.1%) | 94.6%  (90.6% - 99.9%) | 59.9%  (54.4% - 64.1%) |
| **SVM – polynomial kernel** | 0.841  (0.781 - 0.873) | 85.4%  (80.9% - 91.6%) | 36.9%  (32.2% - 40.2%) | 95.9%  (90.9% - 99.9%) | 57.9%  (52.4% - 60.9%) |
| **Neural Network** | 0.828  (0.781 - 0.873) | 86.1%  (82.9% - 92.1%) | 36.8%  (32.8% - 41.3%) | 95.4%  (91.6% - 99.9%) | 58.4%  (53.9% - 63.6%) |
| **Medulloblastoma** | | | | | |
|  | **Accuracy** | **Sensitivity** | **Specificity** | **PPV** | **NPV** |
| **Naive Bayes** | 0.832  (0.757 - 0.875) | 88.0%  (82.5% - 92.7%) | 45.1%  (41.6% - 51.1%) | 96.1%  (91.1% - 99.9%) | 64.0%  (61.3% - 70.5%) |
| **Random forest** | 0.872  (0.801 - 0.944) | 92.7%  (87.5% - 97.2%) | 53.6%  (47.6% - 57.6%) | 97.1%  (92.9% - 99.9%) | 71.5%  (65.8% - 77.5%) |
| **SVM – linear kernel** | 0.830  (0.772 - 0.882) | 88.2%  (82.7% - 94.2%) | 49.3%  (43.6% - 54.8%) | 93.1%  (88.1% - 99.6%) | 68.8%  (64.3% - 74.0%) |
| **SVM – polynomial kernel** | 0.845  (0.785 - 0.877) | 90.0%  (85.0% - 94.5%) | 48.6%  (43.9% - 52.4%) | 95.6%  (89.6% - 99.1%) | 66.3%  (61.3% - 69.8%) |
| **Neural Network** | 0.835  (0.785 - 0.880) | 88.5%  (84.0% - 95.2%) | 48.8%  (44.0% - 55.3%) | 95.9%  (91.1% - 99.9%) | 68.8%  (62.3% - 72.8%) |

Detailed results for different machine learning algorithms for differentiation of the 5 most common posterior fossa tumors among validation datasets. The results are the averaged (95% confidence interval) performance for ×100 repeats 5-fold cross validation (×500 randomly selected validation samples).
